# Vaccination in the Light of the Royal British Commission

**Published:** 1897-11

**Authors:** M. R. Leverson

**Affiliations:** Fort Hamilton, L. I., N. Y.


### VACCINATION IN THE LIGHT OF THE ROYAL BRITISH COMMISSION.

M. R. LEVERSON, M. D., FORT HAMILTON, L. I., N. Y.

(Continued from page 406.)

Mr. Farn was the expert who was to tell all about the lymph. He was examined the 27th of November, and testified that he was appointed in 1871 examiner of vaccine lymph (Q. 4,011), and has examined all tubes of humanized lymph distributed from the National Vaccine establishment; even when he was absent on leave from the office it has been sent to him for examination, and has all been inspected by him (Q. 4,014).

Q. 4,015. "What is the source of the humanized lymph which is distributed from the National Vaccine establishment?" "We derive it from the various public vaccinators throughout the country; some have been specially appointed, and when we have been hard-pressed we have derived it from men whose vaccination was considered to be of high quality."

Q. 4,041. The calf lymph distributed comes wholly from the Board's animal vaccine station in Lambs Conduit Street.

Q. 4,043. "Is the calf lymph supplied exclusively by means of points?" "Except in case where lymph for the vaccination of a calf is required, and then we send it in tubes."

Q. 4,044. "To whom is the lymph distributed?" "To any registered medical practitioner in this country and also to practitioners abroad. Any medical man writing from abroad will have a supply. It is also sent to the Navy Medical Department, and to various colonial officials, civil and military, secretaries and medical officers, and so on."

Q. 4,051. Calf lymph was first supplied in 1881.

Q. 4,053. "Do you recognize any difference between the lymph obtained from the human subject and the lymph obtained from the calf?" "The calf lymph contains blood, I have noticed."

Q. 4,055. It is a rule that calf lymph contains blood. Q. 4,056. You can very often recognize it with the naked eye. It gives a distinct tinge (Q. 4,057).

Q. 4,058. "What is your test for the presence of blood in lymph in extremely minute quantities?" "I determine it for humanized lymph in tubes, by the microscope."

Q. 4,062. "You hold the presence of those (the red blood corpuscles) in the lymph to be a ground of objection?" "My instructions are that it is an objection."

Q. 4,063. I do not know from our reports of any difference of effect between vaccinating from calf lymph and vaccinating from humanized lymph.

Q. 4,064. "From your knowledge would you say that the effects are different?" "I have no knowledge on the point."

Q. 4,065. "What power do you use?" "For the purposes of examination as regards payment an inch, but for other purposes a quarter of an inch."

Q. 4,066. "What power do you use to look for the presence of blood?" "A quarter of an inch. I am not able to use any higher power unless the lymph is blown out."

Q. 4,067. "The presence of how much blood determines you to reject a specimen?" "If I saw a single corpuscle in a tube it should not be sent out."

Q. 4,075. "What do you look for besides blood when you examine under the microscope?" "If the lymph is at all opaque it is placed on one side; or if under the microscope it shows granular bodies the lymph is put on one side."

Q. 4,078. "What else do you look for?" "Extraneous bodies. I have found various things in the lymph. One of the commonest is pearl powder, which, I fancy, they put on the child's arm."

Qs. 4,079-80-81. Dust may have got into tubes, or pieces of a child's clothing. These are recognizable under the microscope, and the tubes containing them are rejected.

Qs. 4,043 and 4,084. Points are used exclusively in the case of calf lymph, and tubes in the case of humanized lymph, and for vaccinating the calf. Points are used for the calf lymph except for vaccinating calves, because the calf lymph coagulates so much that there is difficulty in getting it out of the tubes. He does not know why tubes are used for vaccinating the calves.

Q. 4,099. Witness refers to Dr. Coy for information as to the results of calf lymph and humanized lymph in the hands of the same public vaccinator.

Q. 4,101. Have had statements of undue inflammation from calf lymph, nothing serious beyond that.

Q. 4,102. His records will not permit him to make any statements as to whether there had been undue inflammation from the use of human lymph.

Q. 4,103. (By Mr. Savory). "In perfectly pure,* healthy* lymph what microscopic forms would you expect to see?" "I should hope to see nothing."

Q. 4,112. His method of examination of the lymph within the tube is as trustworthy as he thinks it is possible to be.

Q. 4,119. Blood in calf lymph is not recognized as an objection.

Qs. 4,120-21. There is no charge for any lymph supplied; it is entirely gratuitious even to foreign applicants.

Qs. 4,125-26.—The lymph passes when he approves of it. He is responsible for its purity to the extent of his examination.

Q. 4,127. He is not responsible for failures of the lymph, for one gentleman will fail and another succeed. It depends on the individual vaccinator quite apart from our lymph.

Q. 4,128. (By Mr. Picton). "If it were proved that in

*These adjectives are used by this *eminent* physician in respect to a "lymph" obtained from an ulcer!

any case the lymph was poisonous to the subject of vaccination, would you be responsible for having passed bad lymph as good lymph?" "I have no experience, but I assume that I should be reprimanded if it could be proved in any way that the lymph was not clear."

Qs. 4,131-32. Witness is a microscopist, and it was in a measure as such that he was appointed to his present position.

Q. 4,133. "Have you made any special study of microbes?" "No."

Qs. 4,134-35-36-37. White blood corpuscles are not easily detected in the tubes. Tubes in which he detected either white or red corpuscles would be rejected.

Qs. 4,138-39. He would reject lymph in which there were white corpuscles, because his instructions were so to do, not upon any scientific grounds of his own.

Q. 4,140. (By Dr. Collins). The advantages insured by the microscopical examination is to obviate sending out blood with the lymph.

Q. 4,141. "Are we to understand that blood is the only noxious agency that may reside in the lymph?" "I am only acting up to my instructions as to what I am to reject."

Q. 4,145. "Does evidence exist to show that noxious and untoward consequences from vaccination are referable to the formed constituents of lymph?" "I do not know."

Q. 4,146. "What power do you employ?" "A quarter of an inch."

Q. 4,154. "With such power as you are able to employ would you be able to recognize or distinguish any micro-organisms which might be present?" "No, I should not."

Qs. 4,155-56-57. Although a skilled microscopist, he is not competent to give an opinion as to whether any micro-organisms have been stated to have been identified for erysipelas and so on, but refers to Dr. Klein as a bacteriological authority on the question.

Q. 4,159. "Is there any disease within your experience whose cause you can identify with such microscopical powers as you employ?" "Not that I am aware of."

Q. 4,160. "Can you detect any difference under the microscope between lymph from a healthy child and lymph from a syphilitic child?" "I have never had lymph from a syphilitic child sent me to examine that I am aware of; I have never examined it."

Q. 4,161. "Could you detect it if you had?" "I do not know at all. I never had it to examine, so that I could not tell you."

Q. 4,162. "How do you know that you have never received lymph from a syphilitic child?" "I say so far as I know, of course."

Q. 4,163. "But your knowledge, I apprehend, would only extend to your microscopical inquiries?" "Quite so."

Q. 4,164. "Is it possible to ascertain by microscopical inquiries whether lymph contains the virus of syphilis?" "No; I do not say that it is."

Q. 4,167. "Are you able to recognize any microscopical differences between vaccine lymph and ordinary lymph from an inflammatory surface?" "No, I do not know that I could."

Qs. 4,170-72. Witness had heard of a prize of £1,000 ($5,000) offered by the Grocers' Company for the discovery of any vaccine contagion cultivated apart from an animal body. He did not know whether the award had been made.

Q. 4,173. "Having regard to what you have told us, do you think it would be possible from the microscopical examination you made to guarantee that any lymph was pure?" "No; I should not undertake to say whether it would be a guarantee that the lymph was pure. I do not know that you could do it."

Qs. 4,180-81. Not the witness, but Dr. Cory is the person to apply to with reference to the source of the stock of lymph that was employed for the establishment of the calf station.

Qs. 4,187-88. Witness would be likely to hear of syphilis resulting from the lymph distributed by him, but he has never heard of such an occurrence.

Q. 4,189. "We take every practical precaution we can to insure that the lymph is good."

Q. 4,199. (By Sir Charles Dalrymple). "I thought that where a public vaccinator was in the case you would have to guarantee the lymph that he used in all cases?" "Oh, no."

Q. 4,200. (Dr. Collins). "Are we to understand that as a matter of fact you have ever guaranteed lymph?" "No."

Q. 4,201. (Sir C. Dalrymple). "Then is this inspection that you make of the lymph not a guarantee of the goodness of the lymph?" "I would not say that."

Q. 4,202. (Professor M. Foster). "You guarantee that it is free from such blood corpuscles and such foreign matters as can be seen with such a high power microscope as you can apply to the tube without opening it, and you cannot do more?" "No, I think all practical supervision is made of the lymph to ensure, as far as possible, that it should be free from foreign bodies or from blood."

Q. 4,215. (Professor M. Foster). "Many of the opaque tubes, I suppose, are opaque from the coagulation of the lymph without the presence of blood?" "Yes, no doubt."

Qs. 4,216-17. The rejections solely for blood are about 2 per cent, and from 20 to 25 per cent. for opacity.

Q. 4,233. Witness does not know where the "points" are placed while drying, nor (Q. 4,236) what is done with them after they are charged with lymph. All the charging (Q. 4,239) takes place under Dr. Cory's superintendence.

Q. 4,240. The public vaccinators are paid for the tubes they send up on passing the examination made by witness. (Q. 4,241) The time which elapses between (Q. 4,241) the charging of the tubes and his examination varies very much. The previous week (this was 27th November, 1889) he could scarcely examine any on account of its being so foggy, but as a fair average he examines them within a week.

Q. 4,242. Those left over longest have the greatest percentage of opacity.

Q. 4,243. "How do you explain the opacity? What is the cause of it?" *"That I do not know."*

Q. 4,249. (Professor Foster). "The tube may often be perfectly clear for at least a year, and so far as you know for a longer time?" "Yes."

Q. 4,254. (Mr. Meadows White). "Is there more or less risk of blood finding its way into the lymph in charging a tube, or in charging a lancet?" "I could not tell you."

Dr. Farn's testimony establishes two points: First, that so far as his microscopical examinations are concerned the so-called "lymph" might be teeming with those microbic bodies which it is the fashion or fad of the moment to call the cause of this or that disease without Mr. Farn being able to detect them; and, second, that no guarantee that the lymph is "pure" is ever given by the local Government Board. Beyond this a just review of his testimony might have summarized it by saying that he knew nothing of the least importance. The doing this would have been open to the perfectly just criticism made upon a similar course by the correspondent, an extract from whose letter was given in the number of The Homœopathic Physician for September (p. 348); and for this reason question and answer have been given in their exact words wherever the subject was important. But the scientific reader will not have failed to note the absolute puerility of the laborious work with which the vaccinating officials of the local Government Board amuse their industrious idleness.

The so-called lymph is "pure" according to Dr. Farn, their supreme judge on this question, if there be "no blood" in it, and he determines the presence or absence of this "blood" by the presence or absence of both the red and white corpuscles, the presence of either being, as he says, evidence of the presence of blood. But "lymph"—if it really be the "lymph" which these wise men mean—is the fluid found in the lympathic channels or lymph vascular system, and contains floating in it corpuscles which cannot be distinguished in any way from the white corpuscles of the blood, and, besides this, the lymph stream, reinforced by the chyle which contains similar corpuscles, is poured into the blood stream close to the angle of junction of the right internal jugular and subclavian veins, and thus goes to compose the blood of which it is as essential a part as are the red and white corpuscles.

It is to be regretted that Dr. Collins did not bring out the puerile character of the labors of these officials by a string of questioning which in other instances he proved himself so well able to present for their self-stultification.

